# Identifying the neck margin status of ductal adenocarcinoma in the pancreatic head by multiphoton microscopy

**DOI:** 10.1038/s41598-017-04771-w

**Published:** 2017-07-04

**Authors:** Jian Xu, Youting Chen, Hong Chen, Zhipeng Hong, Zheng Shi, Shuangmu Zhuo, Xiaoqin Zhu, Jianxin Chen

**Affiliations:** 1 Institute of Laser and Optoelectronics Technology, Fujian Provincial Key Laboratory for Photonics Technology, Key Laboratory of OptoElectronic Science and Technology for Medicine of Ministry of Education, Fujian Normal University, Fuzhou, 350007 P. R. China; 2 Department of Hepatopancreatobiliary Surgery, the First Affiliated Hospital, Fujian Medical University, Fuzhou, 350005 P. R. China; 3 Department of Pathology, the First Affiliated Hospital, Fujian Medical University, Fuzhou, 350005 P. R. China; 4 Derpartment of General Surgery, the Affiliated Quanzhou First Hospital, Fujian Medical University, Quanzhou, 362000 P. R. China

## Abstract

Complete surgical resection is the only option for improving the survival of patients with ductal adenocarcinoma in the pancreatic head. After resection, determining the status of resection margins (RMs) is crucial for deciding on the nature of the follow-up treatment. The purpose of this study was to evaluate whether multiphoton microscopy (MPM) could be considered a reliable tool for determining the status of pancreatic neck margins by identifying tumour cells of ductal adenocarcinoma in these margins in the pancreatic head, and our results were affirmative. In particular, MPM could identify tumour cells in the nerves. It was also found that the quantification of the difference between normal duct cells and tumour cells was possible. In addition, the content of collagen could be quantified and used as a marker for differentiating ductal adenocarcinoma in the pancreatic head from normal pancreatic tissues, eventually leading to the identification of R0 and R1 resections of the pancreatic neck margin. With the development of the clinical applications of the multiphoton endoscope, MPM has the potential to provide *in vivo* real-time identification of RM status during surgery.

## Introduction

Pancreatic ductal adenocarcinoma (PDAC) is the most common pancreatic neoplasm and accounts for more than 85% of the total cases of pancreatic malignancy^1, 2^. Most cases of this disease start within the head of the pancreas^3^ and surgery remains the only potentially curative treatment; however, less than 20% of these patients are qualified to undergo surgical resection^4, 5^. The resection margin (RM) status of the patients must be established as an important prognostic predictor. Recent studies have shown that the median survival of patients who underwent resection margin clearance (R0) resection is better than that of patients who undergo a resection margin involvement (R1) resection (see Table 1)^6–12^. Therefore, the correct identification of the RM status is essential to facilitating the long-term survival of the patient.

**Table 1:** Comparison of median survival for R0 and R1 resections.

Study	Year	Study period	Patients (n)	Median survival R0 (months)	Median survival R1 (months)
Wagner[6]	2004	1993–2001	211	20.1	15.3
Verbeke[7]	2006	1995–2003	54	37	11
Raut[8]	2007	1990–2004	360	27.8	21.5
Westgaard[9]	2008	1998–2004	40	15.6	10.8
Campbel[10]	2009	1997–2007	163	25.4	15.4
Gnerlich[11]	2012	1997–2008	285	21.7	16.4
Konstantinidis[12]	2012	1993–2008	1084	23	14

RM is usually performed to achieve a clear margin by obtaining additional pancreatic body parenchyma after intraoperative frozen section analysis (FSA). However, the disadvantage of FSA, which encompasses specimen dissection, tissue sampling and microscopic examination, is that it is time-consuming, because re-resection or re-analysis must be performed if the margin is an R1 resection. Moreover, FSA has reduced accuracy compared to that of haematoxylin and eosin (H&E)-stained histopathology^13^. Hence, a superior method of providing a real-time diagnosis would be highly beneficial to the surgeon and the patient.

MPM is well suited for imaging unstained tissues^14–16^. It can provide detailed real-time information about the tissue architecture and cell morphology based on two-photon excited fluorescence (TPEF) and second harmonic generation (SHG) signals generated by multiphoton excitation^15^. Previous work has reported that MPM could be used to image pancreatic tissues^17–21^, although it has not yet been used to determine the RM status of pancreatic tissues. In this study, our main goal was to close this gap by investigating whether MPM had the potential to identify RM status.

## Results

### Multiphoton images of normal pancreatic tissues

#### Normal pancreatic duct

**Figure 1:**
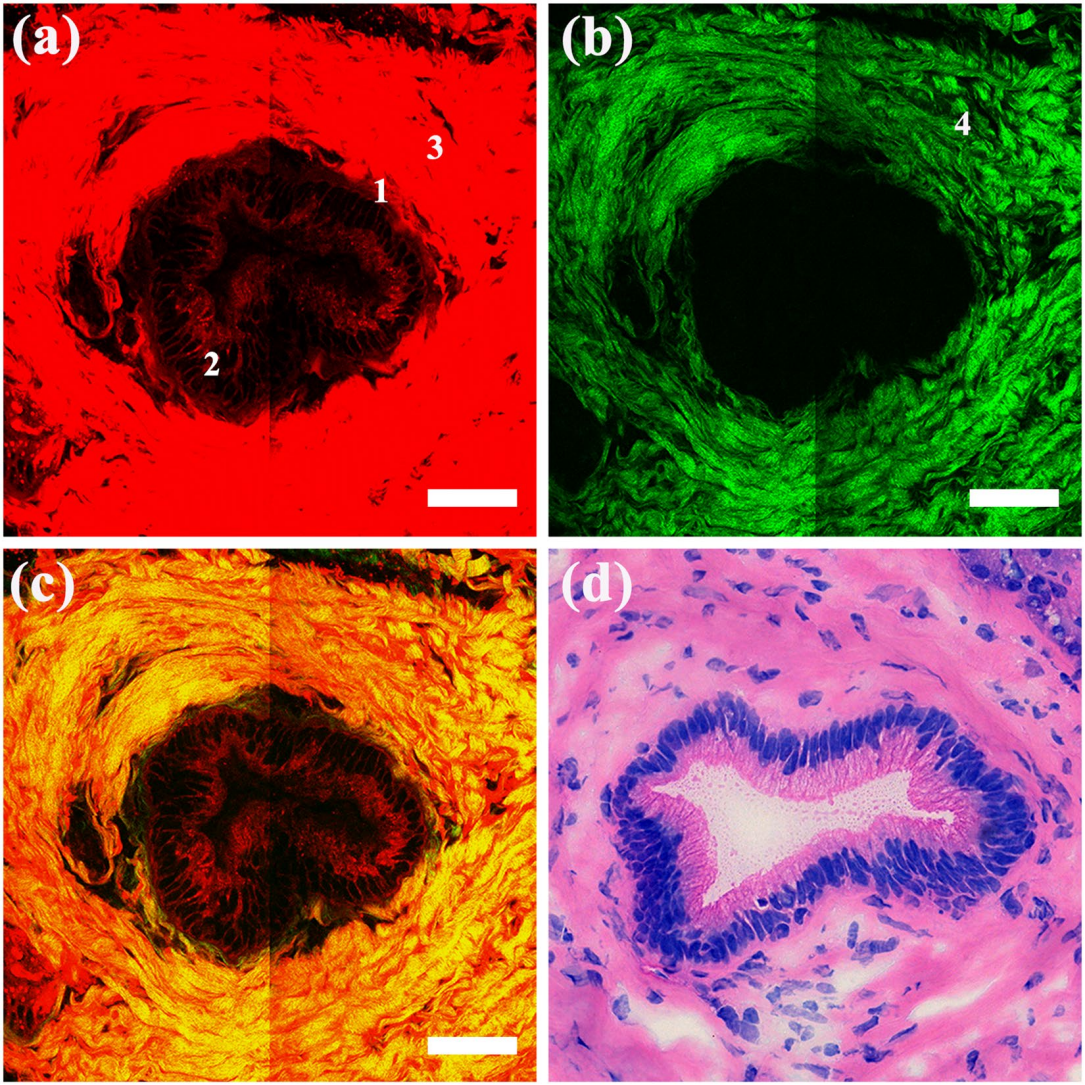
MPM images and the corresponding H&E-stained images of a normal pancreatic duct. Magnification of H&E-stained image is 40 ×; Scale: 50 *μ*m. (**a**) TPEF image; (**b**) SHG image; (**c**) Overlay of TPEF and SHG image; (**d**) H&E-stained image. Position 1: single layer of columnar cells; position 2: multilayer structure of duct cells; position 3: collagen detected by TPEF signal; position 4: collagen detected by SHG signal.

Figure 1 shows MPM images and the corresponding H&E-stained image of normal pancreatic duct. As shown in Fig. 1(a), the collagen around the duct and the individual duct could be detected by TPEF signals. In position 1, the duct presented the typical arrangement of a single layer of columnar cells, and in position 2, presented the multilayer structure of duct cells because of oblique cutting. The nuclei of duct cells faced the stroma with a uniform arrangement and maintained polarity. The fibrous structure of the collagen (position 3) in the stroma was not discernible, but the collagen clusters and the single collagen fibre (position 4) were discernible on the SHG image (Fig. 1(b)), possibly because the cross-link between the collagen fibres can also produce a TPEF signal. According to previous work, collagen can produce SHG signals solely because of its non-centrosymmetric structure, whereas its TPEF signal depends on certain intramolecular and intermolecular cross-links^22^. In the overlay of TPEF and SHG images (Fig. 1(c)), the collagen appears as a yellow substance around the duct, representing the position of the basement membrane. The H&E-stained image shown in Fig. 1(d) is fully consistent with the MPM images.

#### Normal pancreatic lobule

**Figure 2:**
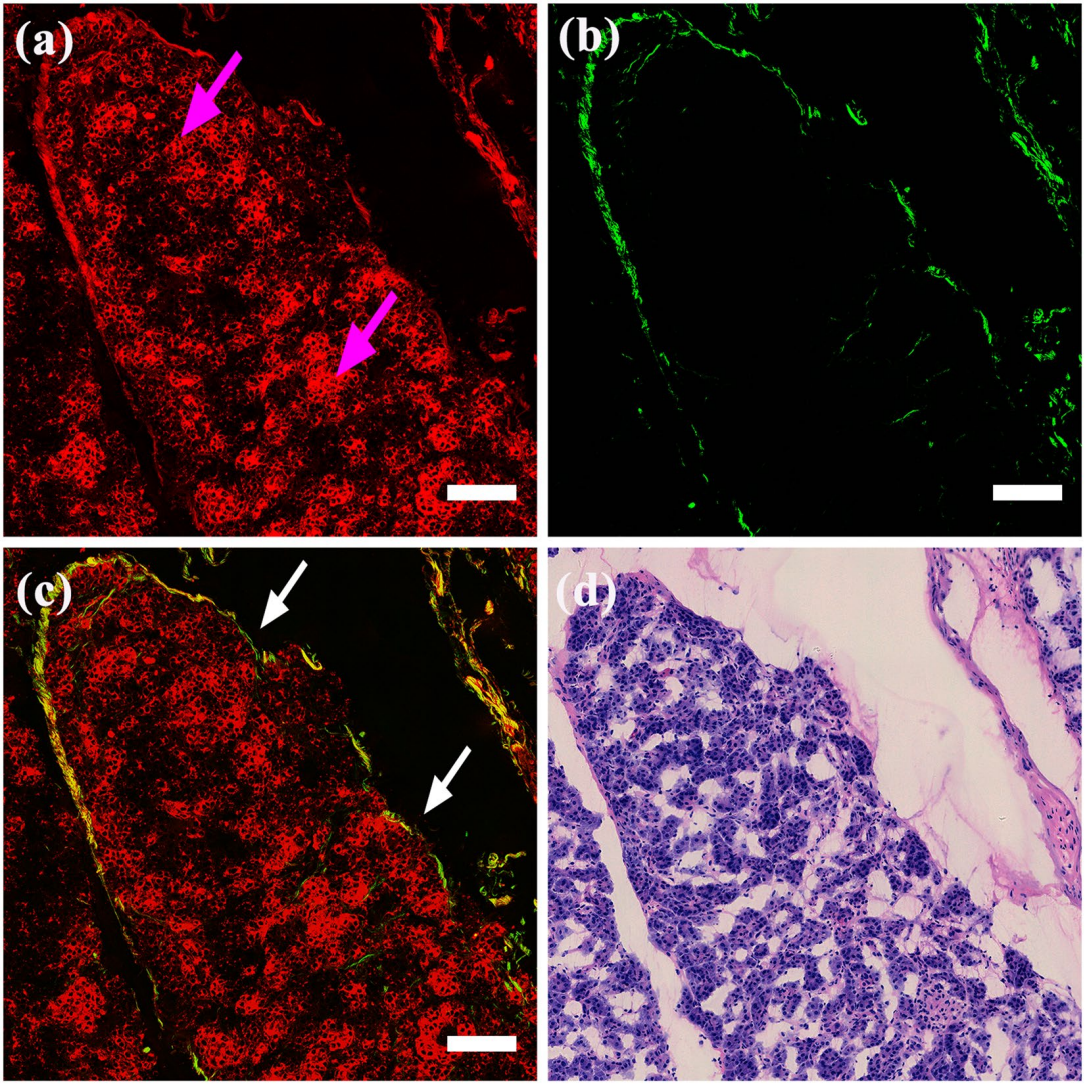
MPM images and the corresponding H&E-stained images of a normal pancreatic lobule. Magnification of H&E-stained image is 40 ×; Scale: 100 *μ*m. (**a**) TPEF image; (**b**) SHG image; (**c**) Overlay of TPEF and SHG image; (**d**) H&E-stained image. White arrows: pancreatic lobule; pink arrows: pancreatic acinar cells.

Typical MPM images and the corresponding H&E-stained image of normal pancreatic lobules are shown in Fig. 2. As shown in Fig. 2(a), the grape-like clusters of pancreatic acinar cells (pink arrows) can be identified. Individual acinar cells are rounded or polygonal with non-fluorescent cell nuclei appearing as dark regions. The ambient cytoplasm could be detected by its TPEF signal. Because the acini were cut obliquely, it was difficult to discern their characteristic shape, which is known as the tubuloacinous gland. However, the acinar cells maintained their regular size with no evidence of cell atypia. The boundary of the lobule and the thin collagen separating the acini could be clearly detected in Fig. 2(b). As shown in Fig. 2(c), the acini and the surrounding collagen formed an integrated pancreatic lobule (white arrows). The H&E-stained image shown in Fig. 2(d) was fully consistent with the MPM images. Due to their sparse population, no pancreatic islets were present on our images. Nevertheless, the main structure of normal pancreatic tissues was identifiable on our MPM images.

### Multiphoton images of ductal adenocarcinoma in the pancreatic head

**Figure 3:**
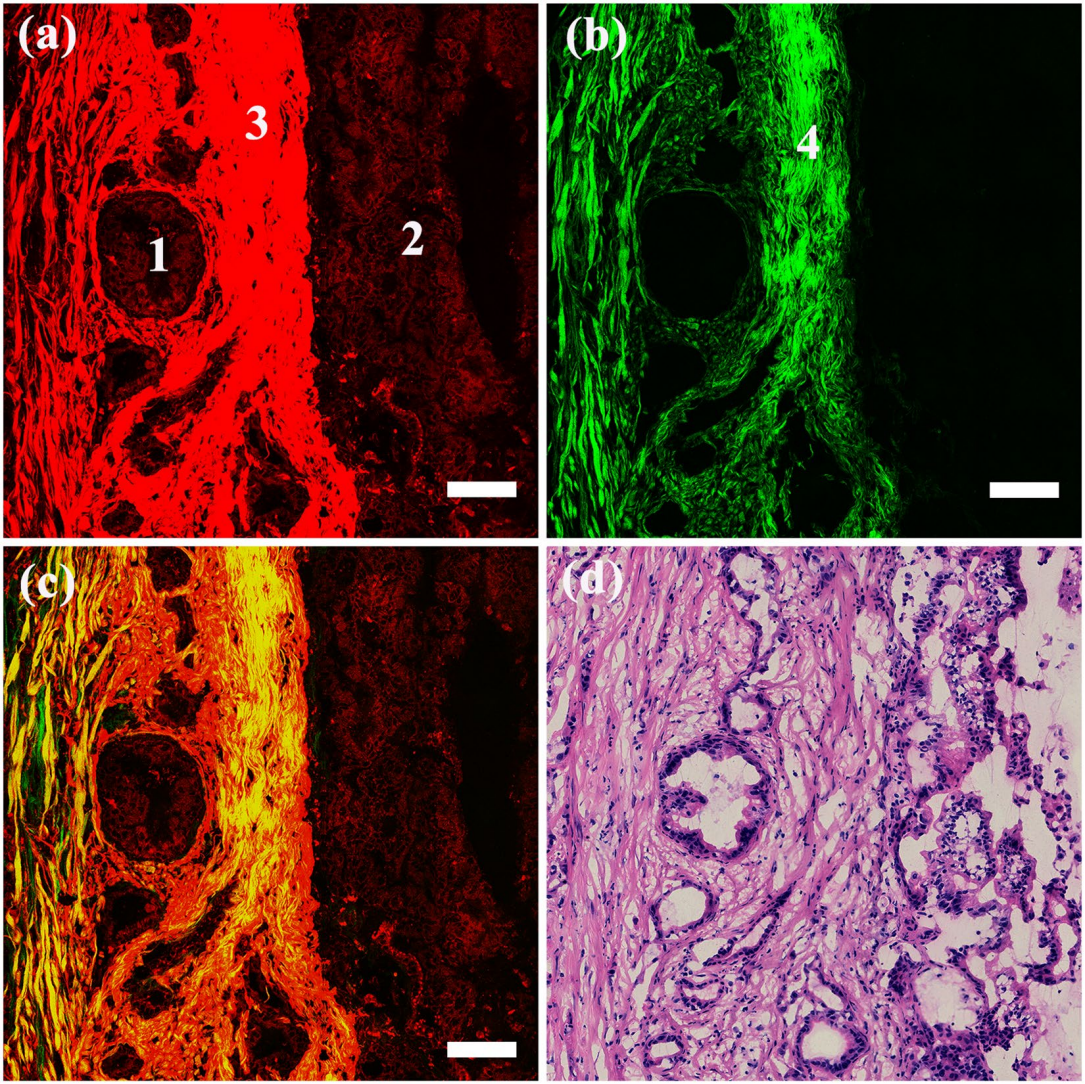
MPM images and the corresponding H&E-stained images of ductal adenocarcinoma in the pancreatic head. Magnification of H&E-stained image is 40 ×; Scale: 100 *μ*m. (**a**) TPEF image; (**b**) SHG image; (**c**) Overlay of TPEF and SHG image; (**d**) H&E-stained image. Position 1: well differentiated neoplastic gland; position 2: poorly differentiated neoplastic glands; position 3: collagen detected by TPEF signal; position 4: collagen detected by SHG signal.

Representative MPM images and the corresponding H&E-stained image of ductal adenocarcinoma in the pancreatic head are shown in Fig. 3. Figure 3(a) shows that the tumour tissue was composed mainly of irregular glands. The variations in the degree of differentiation within the image could be detected. The well-differentiated neoplastic gland (position 1) was embedded in the desmoplastic stroma and showed a duct-like structure, which was more or less similar to the normal ducts. Glands of different sizes could also be detected. Compared to the well-differentiated glands, the poorly differentiated ductal carcinoma (position 2), shown in the left part of this image, was composed of a mixture of irregular glands and tumour cell nests. The complicated, distorted architecture of the poorly differentiated ductal carcinoma rendered the identification of the single gland difficult. The tumour cells in the glands displayed a marked pleomorphic structure, characterized by a variable size and shape. These cells also showed pseudostratification and non-polarity of the nuclei. A significant increase was observed in the content of the stroma, which was simultaneously detected by the TPEF (position 3) and SHG (position 4 in Fig. 3(b)) signals. As previously mentioned, the stroma, which can serve as a growth promoting source of signals, comprised abundant fibrotic tissue^23^. Acinar cells, which were replaced in the process of the desmoplastic reaction, were not found in the ductal adenocarcinoma in the overlay of the TPEF and SHG images (Fig. 3(c)). The H&E-stained image shown in Fig. 3(d) was fully consistent with the MPM images. Together with the normal images, we could morphologically differentiate between normal pancreatic tissues and ductal adenocarcinoma in the pancreatic head.

### Multiphoton images of normal nerve and tumour infiltrating nerve

**Figure 4:**
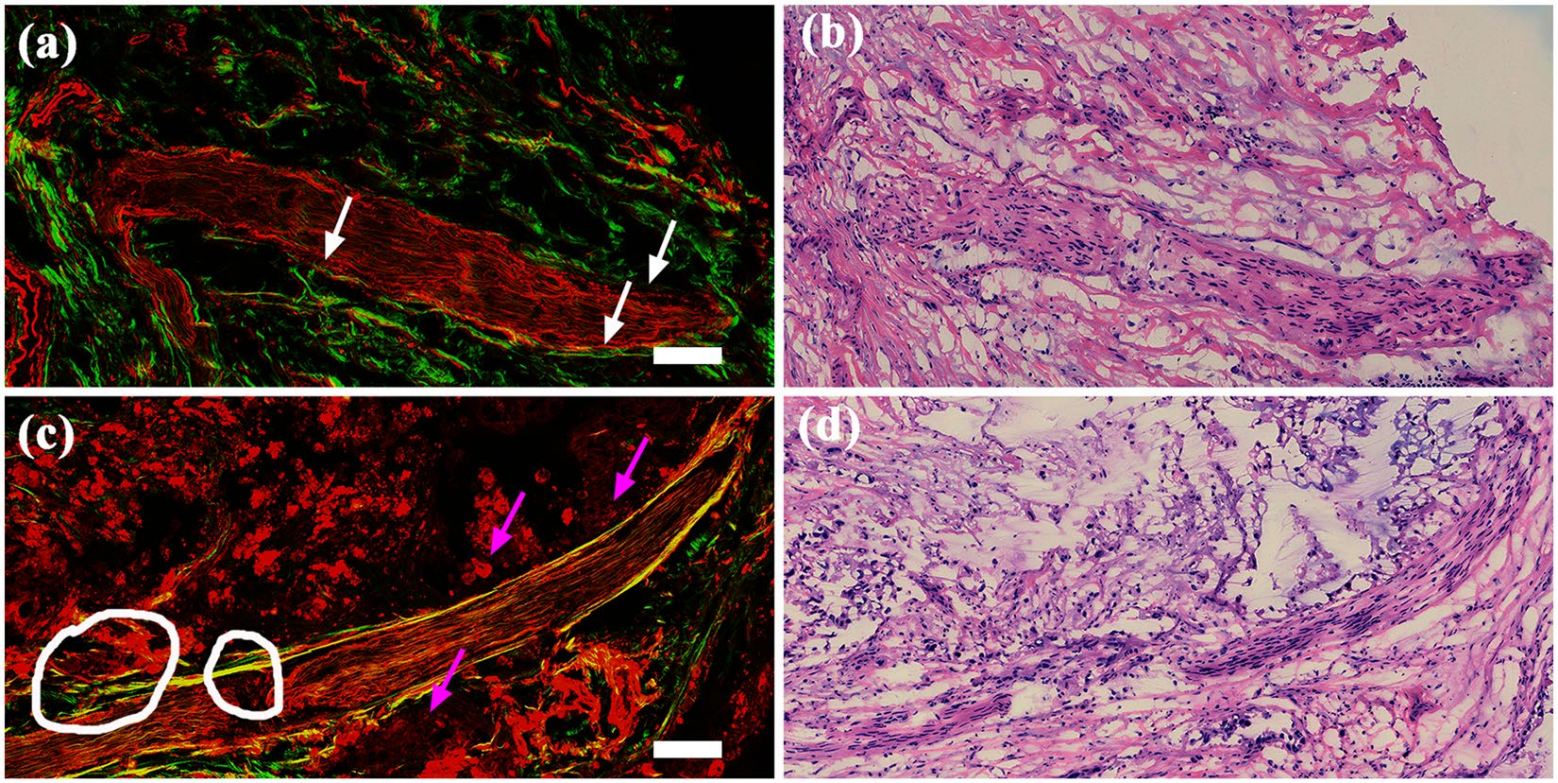
MPM images and corresponding H&E-stained images of a normal nerve and a tumour infiltrating nerve. Magnification of H&E-stained images is 40 ×; Scale: 100 *μ*m. (**a**) MPM image of normal nerve; (**b**) The corresponding H&E-stained image of normal nerve; (**c**) MPM image of tumour infiltrating nerve; (**d**) The corresponding H&E-stained image of tumour infiltrating nerve. White arrows: the epineurium; pink arrows: tumour cells in close proximity to nerve; white circle: tumour cells break through the epineurium and infiltrate into the nerve.

MPM images and the corresponding H&E-stained images of normal nerve and tumour infiltrating nerve are shown in Fig. 4. As shown in Fig. 4(a), the epineurium (white arrows), which is composed of organized collagen fibrils (green colour) and elastin fibres (red colour), binds the nerve fibres into a single nerve and separates this nerve from the surrounding tissue. The nerve sheath, which mainly produces the TPEF signal, could be clearly distinguished from the surrounding connective tissue that was detected by the SHG signal. Perineural invasion is defined as the presence of tumour cells within any of the 3 layers of the nerve sheath or tumour, in close proximity to nerves and involving at least 33% of its circumference^24^. In tumour- infiltrating nerve (Fig. 4 (c)), tumour cells (white circles) break through the epineurium and infiltrate into the nerve. A large number of tumour cells (pink arrows) that are in close proximity to the nerve, can also be detected. The H&E-stained images shown in Fig. 4(b) and (d) were fully consistent with the MPM images shown in Fig. 4(a) and (c), respectively. According to these results, the tumour cells in the nerves can be correctly identified.

### Quantitative analyses of normal pancreatic tissue and ductal adenocarcinoma in the pancreatic head

**Figure 5:**
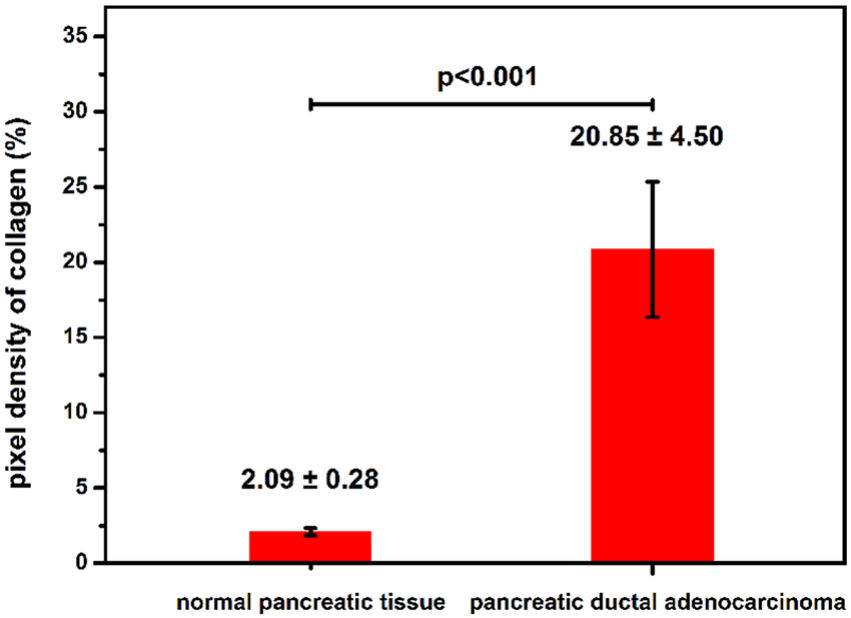
Pixel density of collagen in normal pancreatic tissue and pancreatic ductal adenocarcinoma (n = 8, p < 0.001).

**Figure 6:**
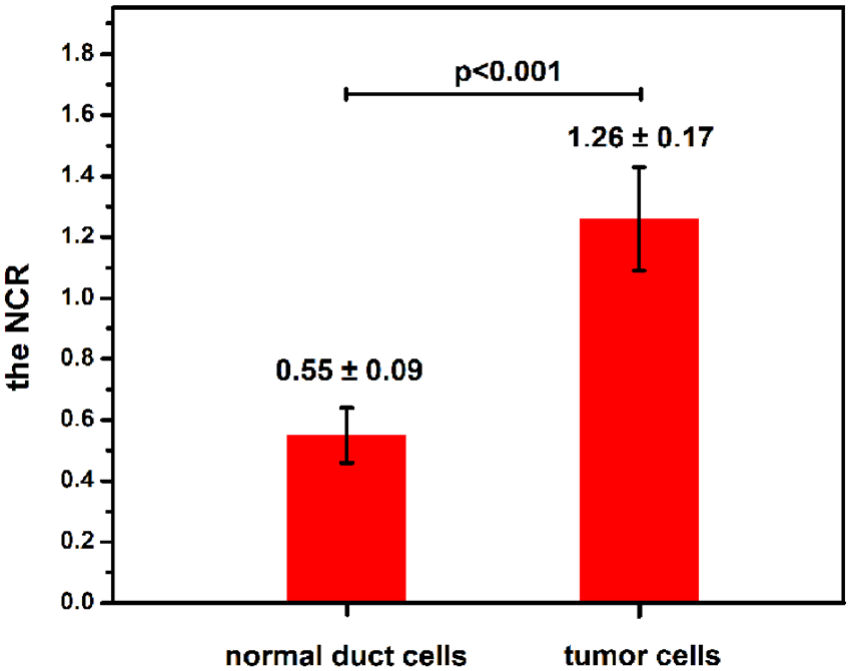
NCR of normal duct cells and tumour cells (n = 30, p < 0.001).

A key characteristic of ductal adenocarcinoma in the pancreatic head is its robust desmoplastic reaction^23^. Most of the tumour volume does not consist of tumour cells, but rather consists of the stroma. The stroma is composed mainly of collagen, which can be observed in our MPM images. Therefore, the content of collagen can be used as a marker to differentiate ductal adenocarcinoma in the pancreatic head from normal pancreatic tissues. To further characterize the difference in the extent of stroma between normal pancreatic tissues and ductal adenocarcinoma in the pancreatic head, the pixel density of collagen was measured. The measurement results showed (see Fig. 5) that the pixel density of the collagen in normal pancreatic tissues was 2.09 ± 0.26 (n = 8) and that in ductal adenocarcinoma of the pancreatic head was 20.85 ± 4.50 (n = 8), clearly revealing that the collagen content in ductal adenocarcinoma of the pancreatic head was significantly higher than that in normal pancreatic tissue. To quantify the difference in cellular morphological features between normal duct cells and tumour cells, the nuclear-cytoplasmic ratios (NCRs) of the normal duct cells and tumour cells were calculated, and the results are shown in Fig. 6. The NCR of normal duct cells was 0.55 ± 0.09 (n = 30), and that of tumour cells was 1.26 ± 0.17 (n = 30); thus, the difference was significant. Therefore, the pixel density of collagen and the NCR of cells can be used as diagnostic indicators to quantitatively evaluate normal pancreatic tissues and ductal adenocarcinoma in the pancreatic head.

### R0 and R1 resections of pancreatic neck margin of ductal adenocarcinoma in the pancreatic head

**Figure 7:**
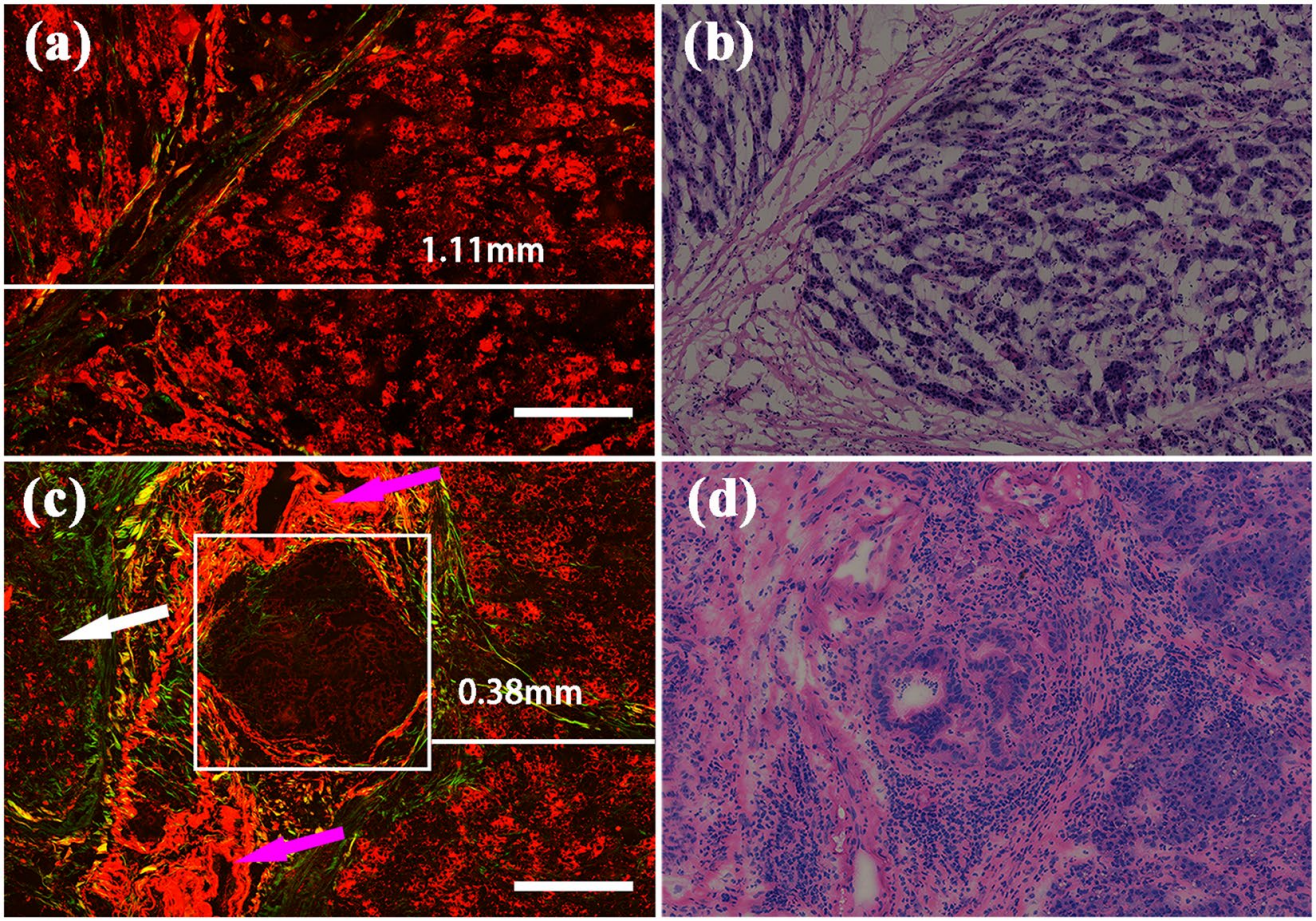
MPM images and corresponding H&E-stained images of R0 and R1 resections of the pancreatic neck margin. Magnification of H&E-stained images is 40 ×; Scale: 200 *μ*m. (**a**) MPM image of R0 resection; (**b**) The corresponding H&E-stained image of R0 resection; (**c**) MPM image of R1 resection; (**d**) The corresponding H&E-stained image of R1 resection. White arrows: increased collagen fibres; pink arrows: blood vessels; white box: neoplastic glands.

Because it is possible to identify tumour cells in nerves and also differentiate normal pancreatic tissues from ductal adenocarcinoma in the pancreatic head, identifying the RM status is easy. The R0 resections and R1 resections of the pancreatic neck margin are shown in Fig. 7. The surgical margin is on the right side of the images. Figure 7(a) shows, in the area 1.11mm from the surgical margin, a finely structured lobule composed of fibrous bundles. Within the lobule, short and thick collagen fibres occurred sporadically, surrounding the acinar cells. However, the acinar cells maintained their regular size with no evidence of cell atypia. This surgical margin was considered the R0 resection. On the image of the R1 resection (see Fig. 7(c)), although the lobule could be identified near the surgical resection, the direct invasion of neoplastic glands (white box) can be observed within 1 mm (0.38 mm distance) of the pancreatic neck margin. The blood vessels (pink arrow) were found near the neoplastic glands. Unlike many solid tumours, the blood vessels that are present in pancreatic ductal adenocarcinoma have been reported to be mostly nonfunctional^25^, whereas the new blood vessels that form in the stromal tissue can help to promote the spread of the tumour cells^26, 27^. Due to the desmoplastic reaction, the acinar cells, located far from the surgical margin, were found to have been replaced by collagen fibres (white arrow). The H&E-stained images shown in Fig. 7(b) and (d) are strongly consistent with the MPM images shown in Fig. 7(a) and (c), respectively. Based on the results obtained from the MPM images, the main characteristics of the R0 and R1 resections were summarized and were presented in Table 2.

**Table 2:** Main characteristics of R0 and R1 resections detected by MPM images.

RM status	Main characteristics that can be seen in MPM images
R0 resection	• No tumour cell is found within 1mm of the surgical margin
	• Normal structures of lobule and/or duct cells are present
R1 resection	• Neoplastic glands or tumour cells are found within 1 mm of the surgical margin
	• Irregular gland(s)
	• Different sizes and shapes of tumour cells
	• Increased collagen fibres

## Discussion

Resection margins are generally believed to be critical to the survival of pancreatic cancer patients^28^. Nevertheless, the reported R1 rates have varied markedly among published works^6, 10, 11^, mainly because of a lack of consensus in the definition of R1. American pathologists use a definition based on the 0 mm rule. The International Union Against Cancer (UICC) defined an R1 resection as ‘the presence of residual tumour cells at the resection margin after treatment’; otherwise, the resection is designated an R0 resection (0 mm rule)^29^. However, in some European centres, the researchers have primarily applied the RCPath criteria in classifying the resection. In this study, we designated an RM as an R1 resection if it had the presence of tumour cells within 1 mm of the RM because there has been growing evidence to suggest that tumour cells within 1 mm of the RM behave more similarly to an R1 resection^12, 30^.

Furthermore, the dissection techniques and tissue sampling also influence the accuracy of R1 rates. For pancreatic tissues, a wide range of dissection techniques is used. A comparison of these dissection techniques emphasizes the advantages of the axial slicing technique, which is based on serial slicing of the pancreatic head on the axial plane^31^. After the specimen dissection, the extent of the tissue sampling also impacts the margin assessment. Due to the highly dispersed and discontinuous growth of pancreatic cancer, it is difficult to identify the microscopic boundaries of the tumour. Thus, R1 rates will be underestimated if the samples are obtained only from the area nearest to the macroscopic tumour. The higher R1 rate obtained from a previous study was based on an axial specimen dissection and extensive tissue sampling^32^.

Interestingly, the R1 rate has started showing a significant increase since a novel standardized pathology protocol came into use^30, 33^. The studies based on this protocol have emphasized how the pathological examination impacts the R1 rate. A pathological examination of an RM involves different steps, but there has again been no consensus or standardization of any of these steps. As a result, the reports on RM status, which is generally believed to be an indicator of the quality of surgery, have been inconsistent. Curative surgery has long been considered the only treatment modality.

These limitations of the existing procedures for assessing RM status necessitate new diagnostic imaging modalities for direct microscopic visualization of the resection margins during surgery. MPM, with a resolution comparable to that of H&E-stained histopathology, can determine the RM status by identifying tumour cells without the aid of any contrast agent. Although MPM remains rare in clinical application, the transformation of this technology in clinics has begun. Researchers first introduced MPM to obtain *in vivo* images of unstained internal organs using a compact and flexible multiphoton microendoscope (MPME) device^34, 35^. The device delivers light from a raster-scanned dual-clad fibre (DCF), which is focused into the tissue by a miniaturized gradient-index (GRIN) lens assembly. The signal from the tissue is collected by the same DCF. The fibre delivery and the small size of this device enable it to be used in clinical endoscopic devices. It is being increasingly used, most recently, by many groups who have developed miniature *in vivo* multiphoton imaging systems for clinical microendoscopy^36, 37^. With developments in multiphoton endoscopy, the intra-operative determination of RM status during surgery should be possible using MPM endoscopy.

In this study, we first used MPM to morphologically distinguish between normal pancreatic tissues and ductal adenocarcinoma in the pancreatic head and quantify the difference between them. The content of collagen was also quantified because it can be used as a marker to differentiate ductal adenocarcinoma in the pancreatic head from normal pancreatic tissues. Additionally, the tumour cells in the nerves were identified. Based on this identification and the differentiation between normal pancreatic tissues and ductal adenocarcinoma in the pancreatic head, R0 resection and R1 resection could be finally detected. These results show that MPM can be used very effectively for the real-time identification of tumour cells and the determination of the RM status of pancreatic neck margins. However, our work has only provided the groundwork for using MPM to determine RM status because the patient specimens used in our study did not encompass the full diversity of patients. Some clinical characteristics of the specimens, including fat content, tumour size, duct dilation, neoadjuvant therapy, and technique of pancreas division, could affect the MPM imaging. To validate the accuracy of RM status determined by MPM, we propose including more patients in our next work. Furthermore, to demonstrate the ability of MPM to determine the margins of the pancreas, we propose focusing our future work on the study of other margins, including bile duct margin, duodenal margins, and retroperitoneal soft tissue margin. With miniaturization of MPM endoscopy, MPM imaging has the potential to provide accurate intra-operative identification of the RM status of ductal adenocarcinoma in the pancreatic head.

## Methods

### Samples

In our study, the pancreatic neck margin tissues of ductal adenocarcinoma in the pancreatic head were provided by the First Affiliated Hospital of Fujian Medical University (Fuzhou, China). The samples, excised from 8 patients undergoing pancreaticoduodenectomy, included 8 pancreatic neck margin tissues. Specifically, the pancreatic necks were divided by knife during surgery because the tissue can be well protected. Once bleeding occurred, the wound was immediately stitched. No examined specimen was divided by Bovie cautery because the margins could be damaged, leading to difficulties in the analysis with this technique. The study was approved by the institutional review board for human research of Fujian Medical University and was conducted in accordance with the Declaration of Helsinki. Written informed consent was obtained from the participants prior to their participation in this study. After surgery, the specimens were placed in a standard pathologic transport container covered with ice and were then sent to the pathology laboratory. Each tissue was sectioned into approximately 7 μm thick specimens using a cryostat microtome. Four of the five consecutive sections were used for multiphoton microscopic imaging, and the middle sample was stained with H&E for a histological comparison with the results of the multiphoton microscopy. To evaluate the accuracy of the MPM results, the samples were also compared to the results of the traditional intraoperative frozen section and final H&E. The results of RM status, obtained from the MPM results, frozen sections and final H&E-stained sections are presented separately in Table 3, along with the clinical characteristics of the specimens.

**Table 3:** Clinical characteristics of specimens and RM status of pancreatic neck margin, as inferred from MPM results, frozen sections and final H&E results.

Sample	Fat content	Tumour size (cm × cm)	Duct dilation	Neoadjuvant therapy	Technique of pancreas division	Operation performed	RM status of pancreatic neck margin		
							MPM results	Frozen section	Final H&E
1	Normal	5.6 × 4.7	No	No	knife	Pancreatoduodenectomy	R1	R1	R0
2	Normal	2.6 × 3.1	Slight	No	knife	Distal pancreatectomy	R0	R0	R0
3	Fatty	2.0 × 1.8	Slight	No	knife	Pancreatoduodenectomy	R0	R0	R0
4	Fatty	4.0 × 3.5	Obvious	No	knife	Pancreatoduodenectomy	R0	R0	R0
5	Atrophic	3.1 × 2.7	No	No	knife	Pancreatoduodenectomy	R0	R0	R0
6	Normal	1.3 × 0.6	Slight	No	knife	Pancreatoduodenectomy	R0	R0	R0
7	Fatty	10 × 7	No	No	knife	Pylorus-preserving pancreaticoduodenectomy	R1	R1	R0
8	Normal	1.5 × 1.16	No	No	knife	Laparoscopic pancreatoduodenectomy	R0	R0	R0

### Instruments

Briefly, the system consisted mainly of an inverted microscope (LSM 510 META; Carl Zeiss, Inc.), equipped with a mode-locked Ti:sapphire laser (Mira 900-F; Coherent, Inc.) and pumped by a 10-W solid-state laser (Verdi-10; Coherent, Inc.). The META detector consists of high-quality, reflective grating and an optimized 32-channel photomultiplier tube (PMT) array detector. It has eight independent channels; each of which covers a spectral width of approximately 340 nm, ranging from 377 nm to 716 nm.

For high-resolution imaging, an oil immersion objective (plan-Apochromat 63 ×, NA = 1.4, Zeiss) was employed. Under the Multichannel Mode setting, two independent-channels were chosen to collect the SHG/TPEF signals. One channel corresponding to the wavelength range of 389 to 410 nm was used to collect SHG signals (green color-coded), and the other channel corresponding to the wavelength range of 430 to 716 nm was used to collect TPEF signals (red color-coded), at an excitation wavelength of 810 nm. The images were obtained at 2.56 μs per pixel, and all of the images were of 12-bit pixel depth.

The light microscope used for imaging H&E-stained sections was a standard bright-field light microscope (Eclipse Ci-L, Nikon Instruments Inc., Japan) with a CCD camera (Nikon, DS-Fi2, Japan). An objective (Plan-flour 40 ×, Nikon) was used for histological examination.

### Margins of interest

**Figure 8:**
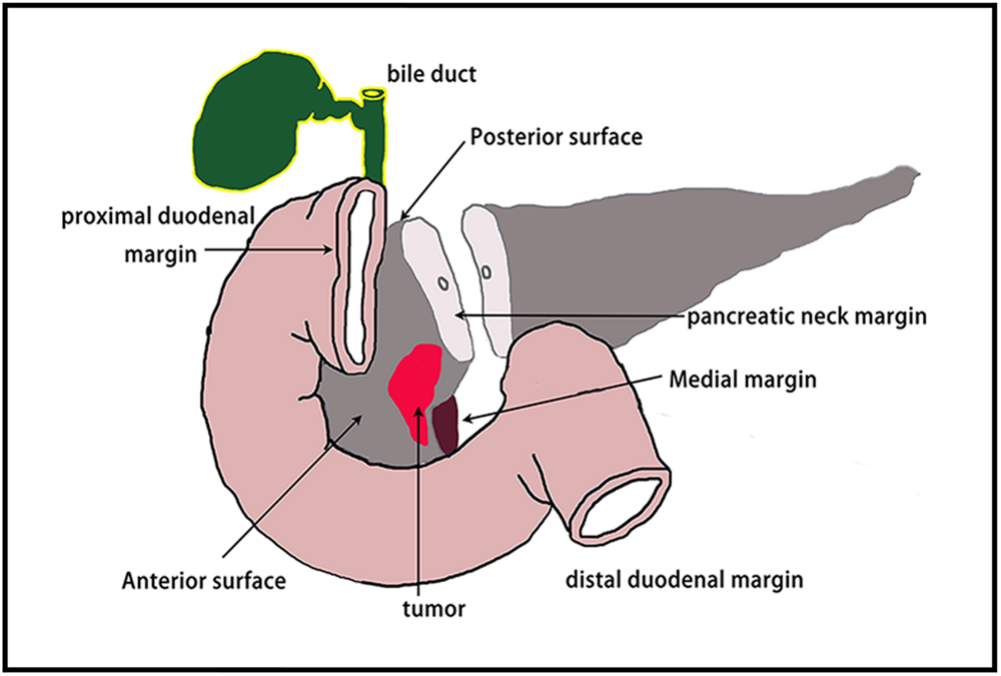
The relevant margins involve the circumferential resection margins and the transection margins of ductal adenocarcinoma in the pancreatic head. The circumferential resection margins include the posterior pancreatic surface, the medial margin and the anterior surface. The transection margins comprise the bile duct margin, the proximal duodenal margin, the distal duodenal margin, and the pancreatic neck margin.

For patients undergoing pancreaticoduodenectomy for ductal adenocarcinoma in the pancreatic head, the relevant margins included the circumferential resection margins (the posterior pancreatic surface, the medial margin and the anterior surface) and the transection margins (the bile duct margin, proximal duodenal margin, distal duodenal margin and the pancreatic neck margin) (see Fig. 8 [drawn by us using Adobe photoshop software]). Generally, bile duct margin, duodenal margins, retroperitoneal soft tissue margin, and pancreatic neck margin must be examined intraoperatively. However, the bile duct and duodenal margins are rarely R1 resections^38^. The retroperitoneal margin, if taken correctly, has a finite extent from the pancreas^8^. The pancreatic neck margin is the most amenable margin to surgical intervention. Of immediate interest to surgeons is whether any additional pancreatic body parenchyma can be taken to achieve a clear margin. Our attempt in this study was, therefore, mainly to identify the RM status of the pancreatic neck margin.

### Histopathologic evaluation

The RM status of MPM was independently interpreted by two trained investigators who had no access to the results of corresponding H&E-stained sections. RM status is used to define the extent of resection in patients with ductal adenocarcinoma in the pancreatic head^39^, and it is classified as either R0 resection or R1 resection. According to the guidelines of the British Royal College of Pathology (RCPath), R1 resection is defined as the presence of tumour cells within 1mm of the resection margin (1mm rule); otherwise, it is designated as R0 resection^40^. The combination of the two investigators’ reviews resulted in the identification of 100% (6/6) of R0 resections and 100% (2/2) of R1 resections, and the same result was confirmed by comparison with the corresponding H&E-stained sections reviewed by an experienced pathologist.

### Quantification methods

**Figure 9:**
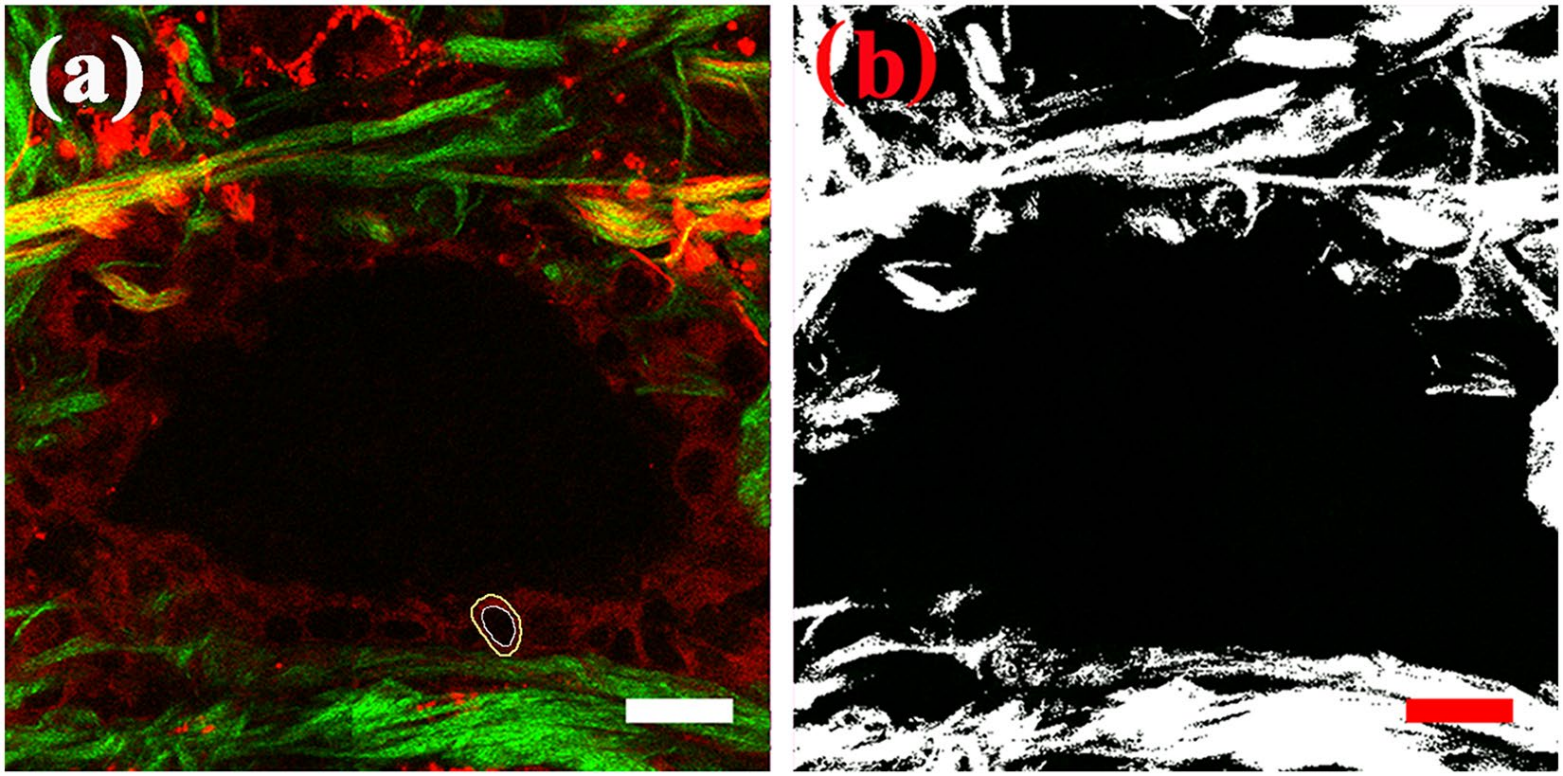
(**a**) Random area of MPM image; (**b**) The corresponding binary image. Scale: 20 *μ*m. White circle: nuclear boundary; yellow circle: cellular boundary.

To describe the changes of normal pancreatic tissues and ductal adenocarcinoma in the pancreatic head, the pixel density of collagen (D_collagen_) and the nuclear-cytoplasmic ratio (NCR) of cells had to be evaluated. For credible quantification of the difference in the extent of collagen, the pixel density of collagen (D_collagen_) was measured using the Histo tool of the LSM 510 system. For example, a random, but typical, MPM image, the total pixel area (A_total_) of which was measured, is shown in Fig. 9(a). The binary image corresponding to this image is shown in Fig. 9(b), the pixel area of collagen (A_collagen_) on which was measured. The pixel density of collagen was calculated by dividing the pixel area of the collagen by the total pixel area, expressed as D_collagen_ = A_collagen_/A_total_.

For cell analysis, the area of the nucleus (A_nucleus_) and the area of the cell (A_cell_) were obtained by measuring the nuclear boundary (white circle) and the cellular boundary (yellow circle), respectively, as shown in Fig. 9(a). The NCR was defined as the ratio of the area of the nucleus to the area of the cytoplasm within the cell, expressed as NCR = A_nucleus_/(A_cell_ - A_nucleus_). The results were calculated using IBM SPSS statistics software and are presented in the form of mean values, followed by their standard deviations (mean ± SD).

### Study steps

To determine the RM status, the correct differentiation between normal pancreatic tissues and ductal adenocarcinoma in the pancreatic head is crucial. Therefore, to demonstrate our differentiation procedure, we first show, on our MPM images, the primary morphological characteristics of normal pancreatic tissues and ductal adenocarcinoma in the pancreatic head. Pancreatic tumour cells reportedly show neurotropism and easily extend along the nerves^41^. Tumour cells are usually discovered in nerves, even if they are not present in the pancreatic parenchyma. Based on these guidelines, the tumour cells in nerves were identified. Then, the quantitative changes in cells and collagen were also described. After identifying the tumour cells in nerves correctly, normal pancreatic tissues were differentiated from ductal adenocarcinoma in the pancreatic head, enabling the detection of R0 and R1 resections of pancreatic neck margin.
